# Evaluation of the national implementation of the VA Diffusion of Excellence Initiative on Advance Care Planning via Group Visits: protocol for a quality improvement evaluation

**DOI:** 10.1186/s43058-020-00016-6

**Published:** 2020-02-27

**Authors:** Monica M. Matthieu, Songthip T. Ounpraseuth, Jacob Painter, Angie Waliski, James “ Silas” Williams, Bo Hu, Robin Smith, Kimberly K. Garner

**Affiliations:** 1 HSR&D Center of Innovation Center for Mental Healthcare & Outcomes Research, Department of Veterans Affairs Medical Center, Central Arkansas Veterans Healthcare System, 2200 Fort Roots Drive, North Little Rock, AR 72114 USA; 2 College for Public Health and Social Justice, School of Social Work, Saint Louis University, Saint Louis, MO USA; 3 College of Medicine, Department of Psychiatry, University of Arkansas for Medical Sciences, Little Rock, AR USA; 4 Geriatric Research, Education and Clinical Center, Department of Veterans Affairs Medical Center, Central Arkansas Veterans Healthcare System, Little Rock, AR USA

**Keywords:** Implementation research, Implementation strategies, Mixed methods, US Department of Veterans Affairs

## Abstract

**Background:**

Traditionally, system leaders, service line managers, researchers, and program evaluators hire specifically dedicated implementation staff to ensure that a healthcare quality improvement effort can “go to scale.” However, little is known about the impact of hiring dedicated staff and whether funded positions, amid a host of other delivered implementation strategies, are the main difference among sites with and without funding used to execute the program, on implementation effectiveness and cost outcomes.

**Methods/design:**

In this mixed methods program evaluation, we will determine the impact of funding staff positions to implement, sustain, and spread a program, Advance Care Planning (ACP) via Group Visits (ACP-GV), nationally across the entire United States Department of Veterans Affairs (VA) healthcare system. In ACP-GV, veterans, their families, and trained clinical staff with expertise in ACP meet in a group setting to engage in discussions about ACP and the benefits to veterans and their trusted others of having an advance directive (AD) in place. To determine the impact of the ACP-GV National Program, we will use a propensity score-matched control design to compare ACP-GV and non-ACP-GV sites on the proportion of ACP discussions in VHA facilities. To account for variation in funding status, we will document and compare funded and unfunded sites on the effectiveness of implementation strategies (individual and combinations) used by sites in the National Program on ACP discussion and AD completion rates across the VHA. In order to determine the fiscal impact of the National Program and to help inform future dissemination across VHA, we will use a budget impact analysis. Finally, we will purposively select, recruit, and interview key stakeholders, who are clinicians and clinical managers in the VHA who offer ACP discussions to veterans, to identify the characteristics of high-performing (e.g., high rates or sustainers) and innovative sites (e.g., unique local program design or implementation of ACP) to inform sustainability and further spread.

**Discussion:**

As an observational evaluation, this protocol will contribute to our understanding of implementation science and practice by examining the natural variation in implementation and spread of ACP-GV with or without funded staff positions.

Contributions to the literature

- Accessing new funding is a discrete implementation strategy used to embed a new evidence-based practice into routine health care settings.
- Few studies in implementation science provide details on the use of financial strategies.
- This VA-funded partnered program evaluation will investigate how dedicated versus detailed staff impacts implementation effectiveness and cost outcomes.

## Background

Traditionally, system leaders, service line managers, researchers, and program evaluators hire specifically dedicated implementation staff to ensure that a quality improvement effort or the integration of evidence-based practices (EBPs) in healthcare settings can “go to scale.” This use of dedicated versus detailed staff to implement programs has relevance and utility within large health care organizations such as the United States (US) Department of Veterans Affairs (VA), in which the use of quality improvement exemplifies a learning health care organization. However, little is known about the impact of hiring dedicated staff and whether funded positions, amid a host of other delivered implementation strategies, are the main difference among sites with and without funding used to execute the program on implementation effectiveness and cost outcomes. Advance Care Planning via Group Visits (ACP-GV), a quality improvement project funded by VA’s Quality Enhancement Research Initiative (QUERI) and the Office of Rural Health (ORH), was developed to address this gap in the literature.

### Use of paid staff as implementation strategy

The use of paid staff to implement a program is a common practice in research and quality improvement cycles within healthcare settings. A recent unpublished systematic review of implementation financing strategies focused broadly on how implementing sites obtained funds. What is rarely reported in detail is how the implementing site spent the money. Therefore, the ability to evaluate funding positions as an implementation strategy to determine the impact on clinical, implementation effectiveness and cost outcomes is novel. Given that most grant mechanisms are predicated on the distribution of funds for the hiring of dedicated staff, the implied or implicit notion is that the funded positions will produce the desired outcomes [1].

### Developing and disseminating a scalable EBP across VA healthcare settings

In 2013, a clinical demonstration project using a novel approach to Advance Care Planning (ACP), a group visit format, was developed by Dr. Kimberly Garner and colleagues in the Veterans Integrated Service Network (VISN) 16 Geriatric Research, Education, and Clinical Center (GRECC). This project, ACP-GV, has garnered acclaim first as an Office of Rural Health (ORH) Promising Practice and now as a Gold Status Practice by the VA Diffusion of Excellence Initiative (DEI). In these ACP-GVs, veterans, their families, and trained clinical staff with expertise in ACP meet in a group setting to have discussions about ACP and the benefits to veterans and their trusted others of having an advance directive (AD) in place

In fiscal year (FY) 2017, the ACP-GV Leadership Team, comprised Dr. Garner and Ms. Taylor, VA’s National Social Work Director, was awarded a multi-year Enterprise Wide Initiative Rural Access Solution grant from ORH. This grant funds an ACP-GV Implementation Team to deploy five implementation strategies to sites to increase ACP engagement with rural veterans. As noted in Table 1, the strategies, organized by the Expert Recommendations for Implementing Change (ERIC) [2] categorization of implementation strategies, includes the name of the implementation strategy, the activity, and the recipient. The five strategies used in ACP-GV are as follows: (1) provide seed funding for new or dedicated staff (e.g., social worker and medical support assistant) who will deliver ACP-GV in rural VHA facilities and community-based outpatient clinics (CBOCs), (2) create a learning collaborative, (3) conduct ongoing training, (4) identify and prepare champions, and (5) conduct audits and feedback against productivity benchmarks using VHA national administrative data. ORH also funds a data manager who analyzes data from ACP-GV monthly site reports and conducts a pilot cost evaluation of staff productivity.

**Table 1:** ACP-GV National Program by ERIC clusters, strategies, activities, and recipients

ERIC strategy cluster category	Implementation strategy	ACP-GV National Program activities developed and tracked by the ACP-GV Implementation Team	Recipient
Utilize financial strategies	Access new funding	ORH funding provided for ACP-GV staff positions in rural VHA facilities and their CBOCs (e.g., social worker and medical support assistant)	Only funded sites
Train and educate stakeholders	Conduct ongoing training	Offer continuing education units to professional clinical staff for attending monthly education calls	Staff at all ACP-GV sites
Train and educate stakeholders	Create a learning collaborative	Promote use of ACP-GV Welcome, Facilitation, and Implementation Manuals on VA Pulse website to staff via weekly administrative calls	Staff at all ACP-GV sites
Use evaluative and iterative strategies	Audit and feedback	Promote use of ACP-GV Implementation Manual on process for documentation, quarterly data collection, and review performance with sites	Staff at all ACP-GV sites
Develop stakeholder interrelationships	Identify and prepare champions	Promote use of VA Pulse website to identify and to deliver materials used to inform local champions and facility leaders on program implementation and sustainability	Staff at all ACP-GV sites

The goal of the ACP-GV National Program is to reach all VA healthcare facilities (*N* = 170). However, the current ORH grant will only reach a portion (*N* = 80) of sites that are considered rural. Therefore, ACP-GV and ORH partners requested our assistance to determine the specific impact of funding staff positions amid the myriad other implementation strategies. This QUERI-funded project has as its main evaluation goal to determine the impact of funding staff positions to implement, sustain, and spread ACP-GV across the entire Veterans Health Administration (VHA).

### What is Advance Care Planning?

Traditional conceptions of ACP have been limited to establishing an advance directive (AD) document. An AD can include both a “living will,” a legal document describing preferences for treatment, and/or a Durable Power of Attorney for Health Care, also a legal document that designates a healthcare proxy to speak for them if they are unable to speak for themselves. However, completing an AD document is only a small part of a larger more complex process. Fried et al. [3] identified key behaviors that are important in advocating ACP as a process instead of an event or just a document to include when completing a living will, designating a healthcare proxy, communicating preferences or guidance, and the importance that the veteran places on quality versus quantity of life with trusted others and clinicians. The ACP-GV program noted here uses this broader conceptualization of ACP.

### Why do ACP in healthcare settings?

The lack of ACP discussions increases the risk that veterans will receive care different from what they prefer and may experience unnecessary interventions leading to increased suffering and higher health care costs [4, 5]. While VHA Handbook 1004.02 mandates personalized ACP discussions including information on AD for all patients in all VHA settings to comply with the Patient Self-Determination Act of 1990 and the Joint Commission, documentation of these discussions in VHA is challenging. This may be due to patient and provider barriers, such as a lack of veteran awareness and lack of perceived clinical priority by providers, respectively [6]. Also, in our clinical experience [7–9], ADs of VHA patients are difficult to quantify using electronic health records or administrative data retrieval systems since they are scanned or hard copy documents. Given these barriers, a new data architecture system launched in FY 20 will provide a national view of ADs across the VHA. Future studies will compare the VA to the community AD completion rate of 26% [10], yet current estimates using this community rate note that 6.7 of the 9.05 million enrolled veterans do not have an AD and may be hospitalized without their medical preferences being known.

### Challenges of Advance Care Planning

Substantial barriers exist that deter optimal ACP. Patients, their families, and healthcare providers often struggle to engage in discussions related to the topics of death and dying [11–14]. Patients often lack awareness or postpone ACP discussions until a crisis requires a life-sustaining treatment decision [15]. VHA Handbook 1004.02 designates the responsibility for ACP discussions to primary care providers. Given that the average primary care visit is about 15–30 min long, providers frequently do not have time during the visit. Although a mandated practice, it may not be perceived as a clinical priority [6]. The innovative approach of groups can also alleviate anxieties of veterans while improving both the quality [16] and efficiency of ACP. The supportive atmosphere of groups can also alleviate anxieties veterans may have when discussing this potentially complex and emotional topic individually with a healthcare provider or significant other. Furthermore, clinical staff members who lead the groups have more time to engage veterans about their preferences and can use the group dynamic to model open dialog with veterans, peers, and trusted others [17, 18].

### Aims

This program evaluation will determine the impact of funding staff positions to implement, sustain, and spread ACP-GV across the entire VHA through the following aims: (1) evaluate the impact of the ACP-GV National Program; (2) among current sites, document and compare funded and unfunded sites on the effectiveness of implementation strategies (individual and combinations) used by sites in the ACP-GV National Program; (3) determine the budget impact of the ACP-GV National Program; and (4) identify the characteristics of high-performing and innovative sites in implementing ACP. If this funding expresses greater participation in the other strategies, then there may be a mechanism of action within accessing new funding alone, or because it is in combination with the other strategies. We will explore this possibility further.

## Methods/design

### Design

**Fig. 1:**
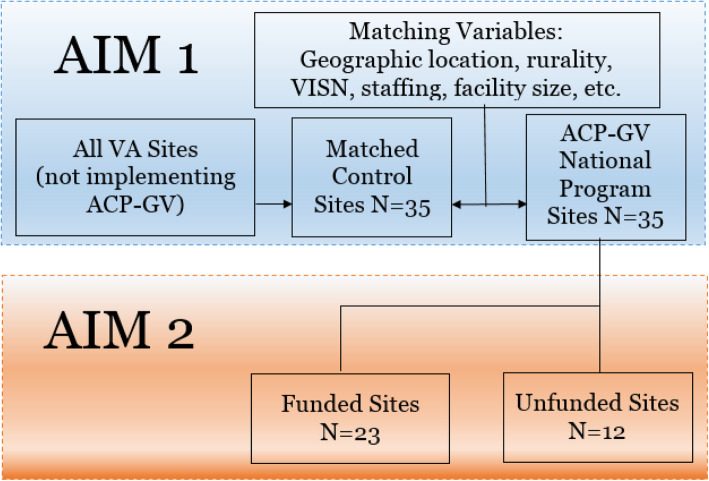
ACP-GV sampling plan for aims 1 and 2

This program evaluation will use mixed (e.g., qualitative and quantitative) methods [19] to determine the impact of the ACP-GV National Program. As an observational implementation evaluation (see Fig. 1), we will start by using a propensity score (PS)-matched control design and VHA administrative data to determine the proportion of ACP discussions in VHA by comparing ACP-GV sites to PS-matched control sites not implementing ACP-GV (aim 1). As a subgroup analysis, we will also determine whether individual strategies or combinations of strategies impact the outcomes of ACP discussion and AD completion rates across the VHA, with special attention to differences between current FY 2019 sites that do (*n* = 23) and do not have (*n* = 12) ORH funding (i.e., DEI-unfunded sites) (aim 2). For aim 3, we will conduct a budget impact analysis of the ACP-GV National Program. Finally, qualitative methods will be used to identify the characteristics of high-performing (e.g., high rates or sustainers) and innovative sites (e.g., unique local program design or implementation of ACP to inform sustainability and further spread) (aim 4).

### Sample

The population for this evaluation potentially includes all veterans who seek care in FY 2019 in VHA (*N* = 9.05 million). The total number of VHA facilities is approximately 170 sites. All veterans who seek VHA services are eligible to receive information on ACP and can complete an AD. Some veterans may choose to engage in an ACP discussion individually while others may elect to attend a group visit. While all veterans are eligible, not all veterans will engage in ACP discussions or ever complete an AD. Using VA administrative data, we will examine all documented ACP discussions, AD completions, and ACP-GV encounters to estimate the sample. In FY 2019, there were 23 funded sites and 12 unfunded sites (i.e., no funding for dedicated staff positions), for a grand total of *N* = 35 sites.

### Data sources

There are three main data sources for this evaluation: (1) VHA administrative data including data obtained from the VA Corporate Data Warehouse, for data at the patient, provider, clinic, and facility levels; (2) ACP-GV Implementation Team Site Tracking Reports, monthly reports from sites on mandatory data elements, Diffusion of Excellence Initiative hub site-level entries, and national data collected on the sites’ participation from initial recruitment to full participation in all of the ACP-GV National Program implementation activities noted in Table 1; and (3) ACP-GV Evaluation Team qualitative interviews, which will collect data on budget impact and implementation consequences, characteristics, and innovations.

The VHA administrative data used here obtains data from the VA Corporate Data Warehouse which includes patient (e.g., name, social security number), provider (e.g., document definition, stop codes, visit date), site (e.g., site name and number), note title (e.g., AD note, AD discussion, AD rescinded, other AD note category), and an ACP-GV-specific workload code. The ACP-GV Implementation Team Site Tracking Report was created specifically to track and to manage the ORH-funded grant activities from recruitment to full-scale implementation as an ACP-GV site. This report tracks all implementation activities offered to any VHA facility that expresses interest in and/or later applies to become a funded or unfunded site. As noted earlier, ORH funding is awarded to rural sites, typically for a social worker and medical support assistant, with the full-time employee equivalent (FTEE) level based on the site’s projected annual workload. The FTEE, amount of funding, and type of positions for staffing of each ACP-GV site is tracked by the ACP-GV Implementation Team in the Site Report.

### Data collection for aims 1 and 2

To complete aims 1 and 2, we will use VHA administrative date from the VA Corporate Data Warehouse data to create patient, clinic, facility, and site-level variables, including veteran demographics, service utilization, AD note titles, and ACP-GV workload. For aim 2, we will merge this data with site-level variables from the ACP-GV Implementation Team Site Tracking Reports, which include sites with funded FTEE and an estimate of the unfunded sites staffing patterns. We will then add to the above data collected on the ACP-GV sites’ participation in the implementation activities from the ACP-GV Implementation Team’s Site Tracking Reports.

For aim 3, we will develop protocols with the ACP-GV Implementation Team to collect new data using an adapted version of the Time Tracking Tool developed by the Behavioral Health Team Based QUERI [20], which will quantify and connect the amount of time each staff member spends in implementation activities to allow for analysis of each strategy alone or in combination. Because implementation costs will be obtained from sites at varying stages of implementation, we will define the index date of transition from pre-implementation to implementation for an individual site as the date of initial use of the ACP-GV-specific workload code and AD discussion note by that site, while sustainability will be assessed using data for the 12 months following the index date when they started implementing ACP-GV. We will reconcile this time tracking data by allocating each cost to a site-specific period (i.e., pre-implementation, implementation, or maintenance) relative to the site-specific index date to use as implementation cost data for aim 3.

## Aim 1: Evaluate the impact of the ACP-GV National Program on the proportion of ACP discussions in VHA by comparing ACP-GV sites to propensity score-matched control sites not implementing ACP-GV

### Design

The 35 ACP-GV sites will be considered “intervention” sites. We will identify a set of matched “control” sites (*N* = 35) defined as VHA facilities not implementing ACP-GV based on an analysis of the ACP-GV workload specific code in VA Corporate Data Warehouse data and not participating in the National Program implementation activities but documenting ACP discussions as noted in the VA Corporate Data Warehouse and using AD note titles. Our hypothesis is that ACP-GV sites (i.e., intervention sites) will have a higher proportion and significantly greater quarterly and annual rates of ACP discussions compared to the PS-matched control sites. Given that the sites were not randomized to either the intervention or control, we will use PS matching to create a “pseudo” randomized evaluation design. The PS is a model-based conditional probability of a site’s membership in the intervention, given its observed site characteristics [21]. We will use a logit model to estimate the PS for each site. The dependent variable of the logistic regression model is being/not being in the ACP-GV intervention group. The independent variables are site characteristics including: rurality, geographic location, Veterans Integrated Service Network (VISN), staffing, facility size, and aggregate veteran demographics such as age, gender, and race/ethnicity. We will use a greedy matching algorithm based on 5-to-1 digit matching [22]. An initial ACP-GV site will be randomly selected from the existing sites to start the process. A non-ACP-GV site (i.e., control) with the closest PS that lies within a fixed distance will be selected for matching. If multiple control sites have PS that are equally close, then one of the control sites will be selected at random. The process will continue until all ACP-GV sites that can be matched have been matched. To evaluate the success of the PS matching, we will compare the balance in the distributions of all the observed site covariates between the two groups using standardized difference plots. An absolute standardized difference of less than 10% suggests a negligible imbalance between the two groups for a given site covariate [23]. A PS matching limitation is if no control sites have PS that lie within the predefined distance (i.e., caliper) of an intervention site, then that ACP-GV site is not included in the PS-matched sample. Given sample size limits, if the PS matching produces a limited number of pair matches, we will consider other strategies, (e.g., PS through stratification, covariate adjustment with PS, or inverse PS weighting).

### Data analysis for aim 1

We will aggregate patient-level outcomes of the ACP discussions and site characteristics (e.g., geographic location, facility size, rurality, etc.) across VHA sites quarterly and annually for descriptive purposes, and then compare the proportion of ACP discussions with the PS-matched control sites. We will specifically examine sites with a high annual rate of ACP discussion and low ACP-GV rates and vice versa to identify patterns and trends. We will present numerical variables as means (SD) or medians (interquartile-range) and categorical variables as counts and percentages (with 95% confidence interval). We will use a significance level of 0.05. To compare annual rates of ACP discussions, the primary outcome for this aim, we will use a two-sided *z*-test with pooled variance to test the difference in proportions across the two groups (i.e., ACP-GV vs. non-ACP-GV). As a secondary analysis, multivariable Poisson regression will examine the differences in annual rates of ACP discussions, while accounting for potential imbalance in site characteristics. Given the sample size, we will build a parsimonious model based on forward model selection.

## Aim 2: Among ACP-GV sites, document and compare ORH-funded and DEI-unfunded sites on the effectiveness of implementation strategies (individual and combinations) used by sites in the ACP-GV National Program on ACP discussion and AD completion rates across the VHA

### Data analysis for aim 2

We will use a comparative case study approach to compare funded sites to unfunded sites on the two outcomes of ACP discussion and AD completion rates for aim 2. Due to the small number of sites, we are hindered in our ability to control for confounders. Yet, we anticipate that additional funding for staff positions will largely impact outcomes. Therefore, we will track the other four implementation strategies (see Table 1) to serve as mediators of impact. If funding expresses greater participation in the other strategies, then there may be a mechanism of action within accessing new funding alone, or because it is in combination with the other strategies.

In our preliminary data, there is some evidence of variation in participation in the implementation strategy of ongoing training by funded sites and increased uptake of ACP-GV vs. unfunded sites. We will use the Implementation Team Site Tracking Report and qualitative interviews from aim 3 and aim 4 to determine if we can make quantitative inferences as to what funding imbues and what impact it has on the other implementation strategies. To provide feedback to our partners for quality improvement and to not lose important trends that may exist in the data that may help understand larger trajectories of change, we will generate quarterly reports tabulating the number and type of implementation strategies used at each site per quarter and the extent to which each of the sites achieved the implementation outcomes. The annual summative evaluation report will describe the implementation strategies that were used by multiple facilities which were most successful and least successful in achieving positive outcomes. These quarterly briefs, annual reports, and our aim 4 analyses will be synthesized to support sustainability and spread of the initiative.

## Aim 3: Determine the budget impact of the ACP-GV National Program

### Design

To accomplish aim 3, we will follow the steps for conducting a budget impact analysis (BIA) [24] developed by the International Society for Pharmacoeconomics and Outcomes Research (ISPOR) [25] and referenced by Smith and Barnett in their discussion of the role of economics in the QUERI program [26]. Implementation- and program-level costs will be collected and analyzed to account for the full impact of the ACP-GV program at individual sites and nationally for VHA.

### Data sources for aim 3

Data will be collected for the pre-implementation and implementation periods to estimate costs associated with ACP. VHA Managerial Cost Accounting (MCA) System data will be used to estimate health care encounter and VHA payroll costs. MCA is a derived database built from standard VHA data sources that allows comparison of cost and utilization characteristics. Activities associated with implementation (implementation period only) and direct provision of the ACP-GV will be collected at the local level and aggregated for the national program. Productivity and time data will be collected using a combination of the VA Corporate Data Warehouse, the Time Tracking Tool, and stakeholder interviews conducted as part of aim 4. Costs will be assigned to these activities based on VHA payroll costs in MCA.

### Data analysis for aim 3

ACP-GV is not expected to have direct short-term impact on overall patient healthcare utilization; therefore, the focus of the analysis will be costs related to implementation and provision of ACP-GV for sites and the National Program. Changes in costs are likely to vary by site depending on the extent to which existing resources are leveraged for provision of ACP-GV. The ability to provide ACP discussions to multiple veterans in a single session via ACP-GV will offset these increased costs; however, the extent to which these two factors exist will vary by site. To estimate direct costs, the time associated with all implementation and program activities will be multiplied by the salary and fringe rate of the team member performing the activity. Each activity will be assigned to a specific time, site, and team member to allow for estimation of costs at the individual site level. Indirect implementation costs (e.g., educational materials) will be tracked and allocated to sites by the ACP-GV Implementation Team.

To examine the budget impact at existing sites, we will compare the costs associated with ACP during the year before and after the site joined the ACP-GV National Program. Cost differences will be analyzed from the payer perspective [24]. For the national program, including all ACP-GV program costs and costs attributed to the implementation strategies identified in Table 1, VHA will be the payer. For program and implementation costs specific to a VAMC, the VA Medical Center (VAMC) budget administrator will be considered the payer. Observable characteristics that are predicted to influence the ACP budget of an individual site will be collected (e.g., VAMC size). Using TreeAge software, a probabilistic sensitivity analysis (PSA) will be conducted [27]. The PSA allows the assignment of a distribution to model parameters rather than just a point estimate to account for uncertainty in the BIA model. To facilitate broader implementation of ACP-GV, a decision analysis model is needed for non-participating sites to estimate the budget impact at their site based on site-specific factors (e.g., VAMC size). The model will be developed in Microsoft Excel, and a user guide will be created and disseminated to sites. This model will show the budget impact at existing ACP-GV sites as well. One-way sensitivity analyses will be possible within the model to allow decision-makers to see the impact of each site-specific factor.

## Aim 4: Identify the characteristics of high-performing (e.g., high rates or sustainers) and innovative sites (e.g., unique local program design or implementation of ACP) to inform sustainability and further spread

### Sampling plan

We will purposively select approximately 24 ACP sites. We will use site reports and our aims 1 and 2 VA Corporate Data Warehouse datasets to identify a total of six sites with high-performing characteristics (i.e., three sites with high productivity in terms of ACP-GV against the performance goal of 200 ACP-GV groups a year/per FTEE and three sites with high rates of AD discussions in the 75th or higher percentile nationally) and six sites that are sustainers after the intensity of implementation strategies begins to reduce (i.e., three sites that meet the ACP-GV performance goal and three sites with high rates of AD discussions). To capture unique site-specific program design or other innovations that might be missed by identifying sites based only on performance, we will identify six sites based on unique local program design characteristics and six with innovative models for implementing ACP known to the ACP-GV Leadership or Implementation Team or from data in the ACP-GV Implementation Team Site Tracking Report.

### Data collection for aim 4

Currently, the ACP-GV National Program and Implementation Team host weekly educational calls for all sites. The Implementation Team routinely tracks training participation at the individual and site level and records it on the ACP-GV Implementation Team Site Tracking Report. Prior to starting aim 4 interviews, we will recruit sites on these calls by describing the opportunity for ACP-GV site personnel who are implementing the program to participate in interviews. We will conduct semi-structured qualitative interviews with the main implementer of ACP-GV at each selected site. These primary points of contact are tracked on the Site Tracking Report as participating in the ACP-GV National Program’s activities. Given that our operational partners deemed this evaluation to be quality improvement and non-research, the ACP-GV Implementation Team will send an e-mail, a copy of an information sheet, and the interview guide to invite the main implementer at the ACP-GV sites to participate. After confirming willingness to participate, a trained qualitative interviewer will conduct the 45-min interview by telephone. To speed the process of transcription, a trained data entry assistant will digitally record this interview using a VHA approved recorder. Interviews will be transcribed verbatim by the data entry assistant (who will also be on the call) and checked against their notes. The interviewer will then prepare an interview report and provide it back to the interviewee for member checking. In order to attain targeted recruitment numbers, the interviewer will work closely together with the ACP-GV Implementation Team in weekly meetings to determine which sites qualify in each category and to discuss the sites’ level of participation. If participation is low, we will ask the ACP-GV National Program to encourage participation verbally on the calls and in their email correspondence with sites.

### Data sources for aim 4

The interview guide will be developed using the Consolidated Framework for Implementation Research (CFIR) Interview Guide Tool and includes the following constructs related to implementation, sustainability, and spread: (1) intervention characteristics: adaptability, cost; (2) outer setting: external policies and incentives; (3) inner setting: networks and communications, relative priority, organizational incentives and rewards; (4) readiness for implementation: leadership engagement, available resources; (5) process: planning; (6) engaging: opinion leaders, reflecting and evaluating. Additional questions to be developed with input from the ACP-GV Leadership and Implementation Teams will focus on ACP-GV specific information on implementation and sustainability, as well as unique characteristics and innovations. Additional questions about the individual and set of implementation activities used, those not used, and rationales for each will be informed by the ACP-GV monthly site reports.

### Data analyses for aim 4

The coding team will include a doctoral trained qualitative interviewer/analyst, a data analyst, and a data entry assistant. We have divided the qualitative data analysis process into four steps: (1) data management: transcripts of the audio-recordings will be entered in Atlas-ti, a software program already on hand that enables analysts to mark blocks of text with codes. Coding will use systematic, iterative, and directed content analysis methods [28, 29]. Upon completion of independent coding by at least two coders, all the transcripts from multiple coders will be merged into one hermeneutic unit. (2) Development of top-level codes: following a deductive approach guided by domains identified from CFIR [30], we will use a two-tiered coding strategy (e.g., top- and sub-level). The team will follow the conceptual framework of the CFIR model and use the interview guide to develop a preliminary code book. (3) Top-level coding: the coding team will independently assign top-level codes to one-half of the transcripts using the preliminary code book, paying attention to themes that relate to performance and sustainability. To ensure coding reliability and consistency, the lead evaluator on the project will review initial and all final coding.

We will also use the Coding Analysis Toolkit (CAT) software to run comparative statistics estimating team consensus in applying top-level codes. If the results do not meet the acceptable reliability criterion of 0.70 or higher, the team will meet to resolve differences. This process will be repeated until coding consistency at or above the acceptability level is achieved, after which the three coders will work independently. (4) Sub-level coding: the team will sub-code top-level codes that are “grounded” (i.e., associated with many quotations). Sub-level coding will follow a similar trajectory to that of top-level coding. After coding is complete, the team will select quotations that illuminate sustainability, innovation, and uniqueness and write summary statements and outline overarching themes across questions and codes, noting any repeating patterns or divergent ideas. Interpretation of the data will compare, contrast, and develop these overarching themes and prepare the summarized data into a final report highlighting variation to include unintended consequences of ACP-GV implementation and sustainability, as well as high-performing and innovative models of ACP. Lessons learned will be drafted from the strengths and limitations of the ACP-GV program. To integrate the quantitative and qualitative data, we will merge data from aims 2 and 4 to refine the existing ACP-GV implementation manual to include sustainability and spread. We will refine the manual in concert with the ACP-GV Implementation Team.

### Trial status

The Institutional Review Board at Central Arkansas Veterans Healthcare System has approved this program evaluation. Data collection for this program evaluation began in October 2019.

## Discussion

This mixed methods program evaluation will produce an impact analysis on the funding of staff positions to implement, sustain, and spread a program, Advance Care Planning (ACP) via Group Visits (ACP-GV), nationally across the entire US Department of Veterans Affairs (VA) healthcare system. To determine the impact of the National Program, we will use a propensity score-matched control design to compare ACP-GV and non-ACP-GV sites on the proportion of ACP discussions in VA healthcare facilities (aim 1). To account for variation in funding status, we will document and compare funded and unfunded sites on the effectiveness of implementation strategies (individual and combinations) used by sites in the National Program on ACP discussion and advance directive completion rates across the VHA (aim 2). In order to determine the fiscal impact of the National Program and to help inform future dissemination across VA, we will use a budget impact analysis (aim 3). Finally, we will purposively select, recruit, and interview key stakeholders, who are clinicians and clinical managers in the VHA who offer ACP discussions to veterans, to identify the characteristics of high-performing (e.g., high rates or sustainers) and innovative sites (e.g., unique local program design or implementation of ACP) to inform sustainability and further spread (aim 4).

This evaluation will rely on quantitative and qualitative methods to evaluate the impact of a set of five implementation strategies deployed by the ACP-GV National Program, primarily through using the CFIR [31]. The strategies are as follows: (1) provide new funding for new or dedicated staff positions who deliver ACP-GV in rural VHA facilities and CBOCs, (2) create a learning collaborative among sites, (3) conduct ongoing training with sites, (4) identify and prepare champions, and (5) conduct audit and feedback with sites using national VA administrative data. There are three main data sources for this QUERI partnered evaluation: (1) the VA Corporate Data Warehouse data base, which contains VHA national ACP and AD data at the patient, provider, clinic, and facility level; (2) ACP-GV Implementation Team Site Tracking Reports, which are monthly reports on ORH mandatory data elements and national data collected on the sites’ participation in the ACP-GV National Program and its implementation activities; and (3) ACP-GV Evaluation Team qualitative interviews, which will collect data on budget impact and implementation challenges, successes, unexpected outcomes, and unintended consequences from ACP-GV implementation. As an observational implementation evaluation, the next steps for this QUERI-partnered evaluation will be to examine the natural variation in implementation and spread of ACP-GV as it rolls out to all VHA facilities across the country.

In order to determine the most effective and efficient delivery mechanism that can be sustained beyond funding staff positions, it is essential to evaluate the impact of funded staff positions as a discrete implementation strategy or if the impact is contingent on the combination with other implementation strategies, on implementation effectiveness and cost outcomes. While this study focuses on the implementation of an EBP in a national health care system within the VA, this program is of enormous value to other health care systems who offer ACP as part of routine service delivery and preparation for health care decision-making with patients and families who may or may not have a surrogate decision-maker identified. Future studies on ACP in other non-VA health care systems need to consider carefully the development of administrative data to capture non-reimbursable services such as ACP and the barriers and facilitators to the use or non-use of ADs on patient, providers, ethics teams, and health care utilization, patient satisfaction, and cost outcomes. Regardless of the program used to conduct ACP, this protocol fills a gap in the evidence base regarding the impact of specifically using funded positions as an implementation strategy and represents unique challenges and opportunities to implementing EBP in health care settings. We anticipate that this program evaluation will provide our operational partners with immediate and actionable information to plan for sustainability when funding for extra staff positions in predominately rural areas is no longer an option. Going to scale with an EBP with or without funded staff positions dedicated to the program is a critical decision-making issue for clinical managers and leaders to consider in mission driven and resources constrained health care systems.

## Data Availability

This paper does not include any data as it is a protocol paper. When data is collected, we ask that readers please request it from the lead author.

## Abbreviations

ACP: Advance Care Planning
ACP-GV: Advance Care Planning via Group Visits
CBOCs: Community-based outpatient clinics
CDW: Corporate Data Warehouse
CFIR: Consolidated Framework for Implementation Research
DEI: Diffusion of Excellence Initiative
EBP: Evidence-based practice
ERIC: Expert Recommendations for Implementing Change
FTEE: Full-time employee equivalent
FY: Fiscal year
GRECC: Geriatric Research, Education, and Clinical Center
MCA: Managerial Cost Accounting
ORH: Office of Rural Health
PS: Propensity score
QUERI: Quality Enhancement Research Initiative
VA: Department of Veterans Affairs
VAMC: VA Medical Center
VHA: Veterans Healthcare Administration
VISN: Veterans Integrated Service Network
